# Editorial: Re-Building and Re-Inventing Workplaces

**DOI:** 10.3389/fsoc.2025.1538872

**Published:** 2025-01-27

**Authors:** Raffaella Valsecchi, Maria Balta, Rachel Morgan

**Affiliations:** ^1^College of Business, Arts and Social Sciences, Brunel Business School, Brunel University London, Uxbridge, United Kingdom; ^2^Faculty of Social Sciences, Business School, University of Kent, Canterbury, United Kingdom

**Keywords:** post-COVID-19 pandemic, re-building organizations, new work structures, flexible working, wellbeing, supportive leadership

During the COVID-19 pandemic, businesses and organizations have had to adapt rapidly to survive, with remote and hybrid work becoming the norm. This shift has highlighted both the benefits of flexible working, such as increased productivity, and challenges like household intrusions and isolation. Organizations are reorganizing practices to manage remote performance and engagement effectively. The rise of digital technologies has facilitated quicker interactions but also revealed gaps, especially in sectors like education, where issues such as inadequate IT infrastructure and reduced face-to-face interaction have emerged. The pandemic has also led to increased sick leave, underscoring the importance of employee health and wellbeing, often overlooked by HR managers. Many businesses have faced significant uncertainty, prompting them to reinvent their operations. This situation offers a chance to rethink workplace structures and practices.

This Research Topic explores how organizations can rebuild and reinvent themselves in response to these challenges, focusing on lessons learned to foster adaptability and success in an ever-changing landscape. The articles for this Research Topic reflect on the necessity of adapting to new work structures and prioritizing employee-wellbeing. The papers include explorations of the need for effective management strategies as businesses shift to remote work settings, while highlighting the importance of reflective practices, continuous training and supportive leadership in challenging times.

Below we summarize these articles briefly, starting with Mustaffa et al.'s study on the effects of communication, training, and transformational leadership during COVID-19 in Malaysia underscores the necessity for businesses to adapt to remote and hybrid work environments. It highlights the challenges of balancing flexibility with issues like isolation and household intrusions. Effective employee engagement strategies are crucial as organizations reshape their structures post-pandemic, emphasizing health and wellbeing. The research calls for reflection on past practices while innovating management approaches. It also stresses the need for improved IT infrastructure to support remote work. Ultimately, the study aims to foster adaptable, resilient workplaces that prioritize employee engagement and wellbeing.

Yang et al.'s research on High Involvement Work Practices (HIWPs) highlights their impact on employee wellbeing and service outcomes during the COVID-19 pandemic. As organizations shift to remote and hybrid work, HIWPs can enhance service performance through customer orientation but may also increase workloads, leading to work-family conflict and reduced wellbeing. The study underscores the need for supportive leadership and the adaptation of management practices to prioritize employee health. Reflecting on past experiences is crucial for developing strategies that balance work demands with employee support, fostering healthier work environments. By integrating these insights, organizations can enhance resilience and adaptability in a changing landscape.

Building on Durkheim's concept of anomic suicide (Ozbilgin et al.) highlight how weakened institutional policies can lead to workplace suicides, especially in the context of the COVID-19 pandemic. As organizations shift to remote and hybrid models, the need for strong support systems becomes critical due to increased isolation and mental health issues. The study identifies dehumanization and misrecognition as key factors contributing to employee wellbeing challenges. It emphasizes the importance of implementing effective practices that acknowledge employee needs and advocates for a proactive approach to prevent work-related suicides. Ultimately, it calls for strategies that foster employee engagement and mental health, creating resilient workplaces.

Exploring equality, diversity and inclusion (EDI) challenges in universities, Toroghi et al. examine the effects of neoliberal managerialism and the COVID-19 pandemic on institutional practices. While remote and hybrid work models have increased productivity, they have also led to isolation and stress, reflecting toxic workplace cultures. The authors argue that a competitive focus on efficiency often undermines inclusivity, exacerbating biases against marginalized groups. They propose the NEAR framework—Noticing, Empathizing, Appraising, and Responding—to cultivate a compassionate workplace culture that prioritizes EDI. In the post-pandemic landscape, fostering organizational compassion and inclusivity is essential for building resilient, supportive environments that enhance employee engagement.

Lastly, Díaz et al.'s exploration of ongoing professional training for social education practitioners underscores the urgent need for adaptability amid societal changes, especially during and after the COVID-19 pandemic. As organizations shift to remote and hybrid models, challenges arise in fostering effective practices. The study highlights the importance of continuous training in mental health, management, and mediation. The pandemic has revealed vulnerabilities in traditional work structures, emphasizing health and wellbeing. Both business and education sectors must reflect on past experiences to re-imagine their practices. A compassionate, ethical approach can foster supportive environments, enhancing individual wellbeing and organizational resilience in serving diverse communities.

Utilizing diverse conceptual and methodological perspectives, the articles in this Research Topic emphasize the need to adapt to new work structures during challenging times. They highlight the importance of employee wellbeing and equality, diversity, and inclusion (EDI) while examining effective management strategies for businesses transitioning to remote work environments.

